# Impact of the Geometrical Parameters of Dolomite Coarse Aggregate on the Thermal and Mechanic Properties of Preplaced Aggregate Concrete

**DOI:** 10.3390/ma13194358

**Published:** 2020-09-30

**Authors:** Agata Stempkowska, Tomasz Gawenda, Zdzisław Naziemiec, Krzysztof Adam Ostrowski, Daniel Saramak, Agnieszka Surowiak

**Affiliations:** 1 Faculty of Mining and Geoengineering, AGH University of Science and Technology, Mickiewicza 30 Av., 30-094 Krakow, Poland; stemp@agh.edu.pl (A.S.); gawenda@agh.edu.pl (T.G.); dsaramak@agh.edu.pl (D.S.); asur@agh.edu.pl (A.S.); 2 Glass and Building Materials Department, Institute of Ceramics and Building Materials, Research Network Łukasiewicz, 31-983 Krakow, Poland; z.naziemiec@icimb.pl; 3 Faculty of Civil Engineering, Cracow University of Technology, 24 Warszawska Str., 31-155 Cracow, Poland

**Keywords:** concrete, regular and irregular aggregates, thermal and mechanical properties, CT analysis, porosity

## Abstract

The article shows investigations on the behavior of preplaced aggregate concrete with regular and irregular coarse aggregates. The thermal properties, compressive strength, and internal structure were analyzed based on computed tomography images. The regular and irregular shapes of aggregates were obtained according to patented technology, which is possible to produce in both laboratory and industrial conditions. Based on the conducted calculations, heat storage capacity was assessed. The influence of grain shape on the material strength, porosity, and hydration gaps was determined. Debonded porosity, as a result of aggregate impurities, was shown using computer tomography analysis. It was shown that the arrangement and shape of the grains has a significant impact on the performance properties of hardened preplaced concrete.

## 1. Introduction

Different types of concrete are widely used in all types of civil engineering constructions. Approximately 70% of its volume is aggregate, and therefore, the quality of the aggregate significantly affects the properties of the concrete mix, such as consistency, workability, and water demand, as well as the properties of the hardened concrete e.g., strength, waterproofing, water absorption, and frost resistance [1]. The main purpose of the aggregate in concrete is to create a kind of “stone scaffolding” in its internal structure that tightly fills the material volume. This is necessary in order to minimize the cement consumption that is needed for bonding. When creating this “scaffolding”, the following principle should be taken into consideration: the ratio of coarse to fine aggregate is higher when the grain size of the fine aggregate is finer. [2]. Too large amounts of fine grains increase the demand of the whole system for cement and water, which may, apart from economic reasons, also lead to a decrease in some of the utility parameters of hardened concrete. Properly selected grain size is a composition of various aggregate fractions with an optimal crumb stack, usually consisting of fine aggregate and several types of coarse aggregate. Granulation should be of adequate continuity in order to ensure the tightness of the crumb concrete mix. Fine aggregate (sand) performs the stabilizing role of the concrete mix, affecting its workability, viscosity, and susceptibility to segregation, while coarse aggregate (gravel, grits) is a volume filler [3]. The quality parameters of aggregates, including the shape of grains, are extremely important in this process [4]. The geometrical shape of the aggregate determines the basic parameters of the concrete and also the properties of the concrete mix. Until now, research has been conducted based on the texture of smooth and rough grains and their sphericity, i.e., rounded, crushed [5,6], as well as their lithology [7] or chemistry [8,9]. With regard to the chemical aspect, the influence of migrating substances (especially chlorides) and their precipitation in the pores and microcracks of aggregate were studied [10]. The scientific work also emphasizes the impact of the method of aggregate processing [11]. The shape and geometry of grains are sometimes analyzed using numerical methods and imaging techniques [12,13], and the aggregate behavior during loading is also examined, especially in terms of arising microcracks [14]. In the literature, there are also attempts to replace natural aggregates with their recycling counterparts, such as crushed concrete, bricks, asphalt [15], or steel slag [16]. The hydration processes and strength parameters of such concretes are also examined [17]. 

The preplaced aggregate concrete (PAC) is a characteristic and untypical type of concrete, which is different than concrete performed in the traditional manner. The main differences relate to the raw material’s characteristics, preparation process, and raw materials content [18]. PAC could be considered for use in civil engineering, especially in prefabricated structures. According to Lv et al., PAC could be adapted as a filler of composite structures such as Concrete Filled Steel Tube Columns (CFSTC). This type of element could be used in underground structures, high-rise buildings, and long-span bridges [19]. According to Alfayez et al., highly eco-efficient PAC due to special characteristics such as its impact resistance and high tensile strength could be useful in infrastructure applications—for example, the design of pavements [20]. In recent years, roller compacted concrete, containing similar ingredients to conventional concrete, has been recommended in pavement construction projects due to the lower possibility of thermal cracking [21]. It has been proven that the volume and type of aggregates used in concrete mixtures have a major impact on its conductivity [22]. Considering the above, it should be stated that the aggregate morphology can significantly affect the behavior of PCA.

## 2. Research Significance

In the design process of concrete, relatively little attention is paid to the geometry of aggregate. Most research works focus on cement binder modifications while looking for new additives and admixtures to improve the quality of concrete. In the work cited above, Rocco et al. studied the impact of shaped (spherical) grains of artificial aggregate made of sintered mullite on concrete properties, i.e., tensile strength, susceptibility to cracking, modulus of elasticity, etc. Artificial aggregate production enables grain sphericity to be easily obtained. Until now, it has been problematic to obtain natural crushed aggregates of a regular shape. The technology developed by AGH University of Science Technology makes it easy to obtain cubic or flat-shaped aggregates. Admixtures and additives are usually difficult to use, and any change in the chemical composition of the clinker requires corrections and refinement of the recipe. There are not many studies on aggregate itself, and the research is limited to the impact of rock chemistry on the quality of the aggregate–binder combination [23]. Research on the impact of shape comes down to computer modeling, which is why the authors decided to look at this problem in an empirical way.

## 3. Materials and Methods

### 3.1. Preparation of Dolomite Aggregate

**Figure 1:**
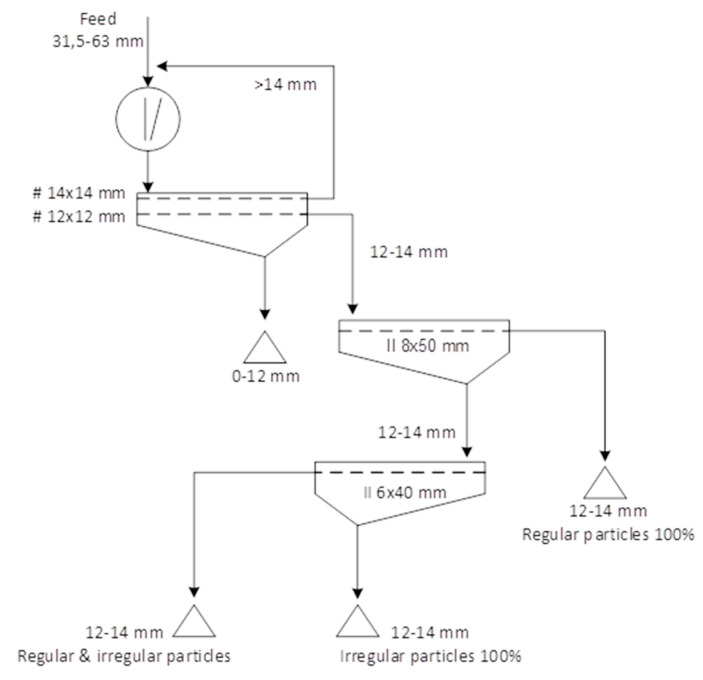
A schematic diagram of the technological system according to the invention [25].

**Figure 2:**
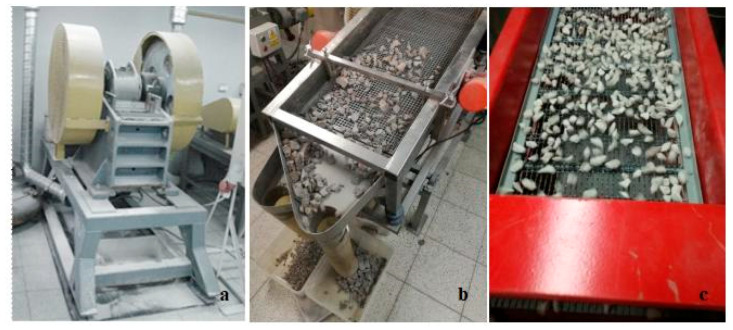
Jaw crusher (**a**), double deck vibrating screen (**b**), and vibrating screen with slotted sieves (**c**).

**Figure 3:**
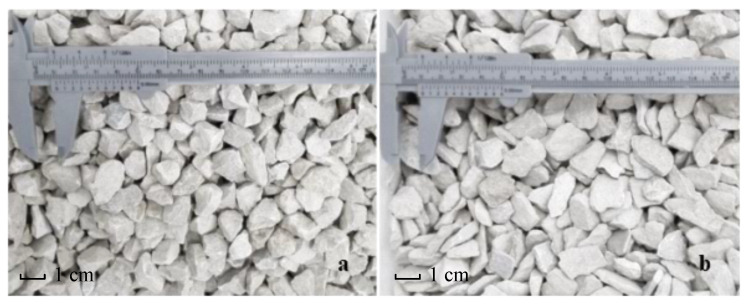
Regular aggregate grain size of 12–14 mm (**a**), and irregular aggregate grain size of 12–14 mm (**b**).

The aggregate used in the study is dolomite from the Imielin deposit (Kopalnia Dolomitu sp z o.o., Imielin, Poland). Due to their basic chemical nature, dolomite aggregates are a good mineral filler in simple concretes [24]. The production of the crushed aggregates involved the mechanical processing of dolomite based on a patented technology for producing a designed shape of aggregates [25]. The technology enables any narrow granular fractions to be obtained separately, with the content of regular and irregular grains reaching 100%. Simplified diagrams of the technological system for the production and processing of mineral aggregates are presented in Figure 1. The system consists of crushing the raw material in a crusher and then classifying the aggregates into narrow grain grades in a screen. Each narrow grain class was separated during a screening process on screens with rectangular (longitudinal) meshes in order to obtain regular and irregular grains separately. In the production of the aggregates for this study, dolomite with a grain size of 31.5–63 mm was ground in a jaw crusher (Makrum, Bydgoszcz, Poland) (Figure 2a). The product was screened on a double-deck screen (Figure 2b) to isolate the 12–14 mm grain size class. The oversized product was recycled for crushing. The 12–14 mm grain size class was screened on a second vibrating screen (Figure 2c) with an 8–50 mm mesh to separate regular grains (upper product of the screen) (Figure 3a). The lower product was separated on the third screen with a 6 mm × 40 mm mesh in order to separate the irregular grains (Figure 3b). A wide description of research concerning the production process of regular and irregular aggregates is provided in the literature [26,27]. The shape of the grains was assessed on slotted sieves according to the standard PN-EN 933-3:2012: Tests for geometrical properties of aggregates—Part 3: Determination of grain shape using the flatness indicator [28].

Properties of dolomite aggregate. Dolomite aggregate can be used as an alternative to gravel aggregates in the production of simple concrete, as well as for floor, contractor, or hydrotechnical concretes. This is due to similar functional properties such as resistance to grinding and abrasion, resistance to crushing, frost resistance, water absorption, and the rheological properties of the concrete mix [29,30]. For the purpose of this work, a specific density of 50 grains was determined by using the pycnometric method in accordance with the PN-EN 1097-7:2008 standard [31], with water absorbability being determined according to the PN-EN 1097-6:2013 standard [32]. Table 1 summarizes the average values of grain density and absorbability, as well as the values of standard deviations from the average values. A higher value for water absorbability was observed in the case of irregular grains, which directly translates into a bigger porosity and frost resistance of the aggregate. A higher standard deviation indicates a greater heterogeneity of the material. The density of regular and irregular grains remains substantially homogeneous.

### 3.2. Concrete Mix

The tests were carried out on 21 different concrete samples, with dimensions of 100 mm cubic sides, made of regular and irregular dolomite aggregates in the 12–14 mm class. In order to determine the compressive strength of concrete, 18 samples were tested, while the remaining ones were subjected to thermal tests. Preplaced aggregate concrete is prepared by placing coarse aggregate into formworks and then injecting the grout mortar into the void between the coarse aggregate [33]. The composition of the mix is presented in Table 2. As a binder, a standard CEM I 42.5 R clinker was used, the main components of which are Portland clinker (95%) (chemical composition CaO–63.94%, Fe_2_O_3_–2.75%, SiO_2_–20.26%, Al_2_O_3_–4.89%, others–8.16%) (Górażdże Cement SA, Opole, Poland) and a setting time regulator (up to 5%). No additives and chemical admixtures were included, as the research focused on the properties of aggregate, specifically coarse fractions of 12–14 mm with various shapes. Moreover, the mixes were not prepared using a continuous graining curve. The reason for this was to capture the effect of the coarse grain shape on the material properties as much as possible, which is the main purpose of the research presented in this article. In this way, basic samples were obtained, and potential factors that could affect the test results were eliminated. Therefore, the aggregate shape remained the only variable. As a result, the water-to-cement ratio was equal to 0.62. Thanks to this, the appropriate workability of the cement matrix that was added to the aggregate was ensured.

**Figure 4:**
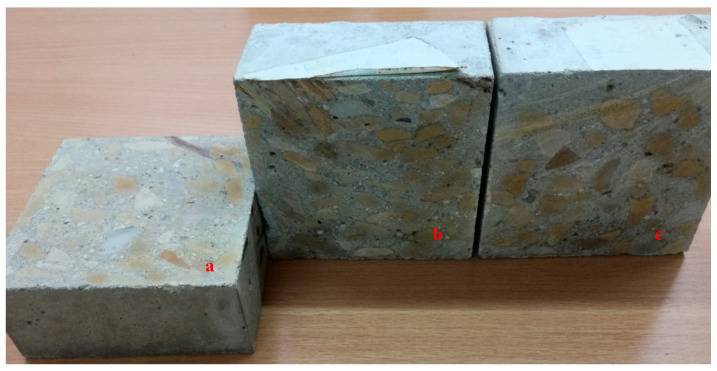
Concrete samples tested: irregular grains 100%—cutting the cube parallel to the grains (**a**); irregular shape 100%—cutting the cube perpendicular to the grain position; (**b**) and regular grains 100% (**c**).

Twice as many cubes were made of the concrete mix with irregular aggregates, which were cut parallel and perpendicular to the pouring direction for the purpose of the thermal and structural tests. The cubes of regular aggregates were cut perpendicular to the pouring direction (Figure 4) In the case of cubes with regular aggregates, the cutting direction does not matter, because there is no anisotropy of the aggregate arrangement.

When preparing the test mixes, differences in flow were noted, i.e., in concrete consistency. In the case of the shaped aggregates, the measured flow is greater and equal to 175 mm, and the consistency is smaller, which confirms the lower demand for water and cement mix. The flow was 150 mm for the irregular aggregates. Similar results were obtained in studies on the formation of fibro concretes from regular and irregular aggregates [34].

After 28 days of the concrete aging, the samples were subjected to strength and thermal tests using a thermal imaging camera. The difference in the cooling temperature of the concrete with regard to the type and arrangement of aggregates was determined, as well as the calculation of the thermal parameters (heat quantity, heat power, heat accumulation capacity). Then, the concrete samples were subjected to internal structure tests using a GE tomograph in order to determine air pores and hydration cracks. 

### 3.3. Research Methodology

#### 3.3.1. Thermal Tests

Thermal tests were carried out using the NEC ThermoGear G100 thermal imaging camera (NEC Avio Infrared Technologies co., Ltd., San Fernando, CA, USA), which enables the registration and visualization of temperature distribution on the surfaces of objects (mapping of the thermal image of objects). The camera works by converting infrared radiation emitted or reflected by these objects into an electrical signal and then into an image viewed on the screen—the so-called thermogram. Thermovision allows the detection of many properties of plastics in a way that no other technology can. The camera was placed at a distance of 40 cm from the tested sample. The samples were placed in a laboratory dryer that was heated to 160 °C for 2 h. After this time, the temperature was measured to determine the cooling time. At the beginning of the measurement, the temperature between consecutive measurements was measured every 30 s, then every minute, and later at longer intervals.

On the thermal imaging camera, a program can be set for reading temperature values on the surface of samples in various places. To calculate the heat capacity, the value *ave* was used, i.e., the average temperature collected from the entire surface of the sample. Thermal properties of concrete materials are attracting increasing attention, not only because of their effects on building energy efficiency but also on structural performance and serviceability. More and more often, modern concrete materials containing various types of aggregates (lightweight, natural recycled aggregates, with regular and irregular grains) and fibers are used. These materials are used in transport structures such as pavements and bridges platforms as well as large foundations (bulk concrete) where thermal behavior is important and sensitive to the behavior of the structure.

#### 3.3.2. Strength Test

**Figure 5:**
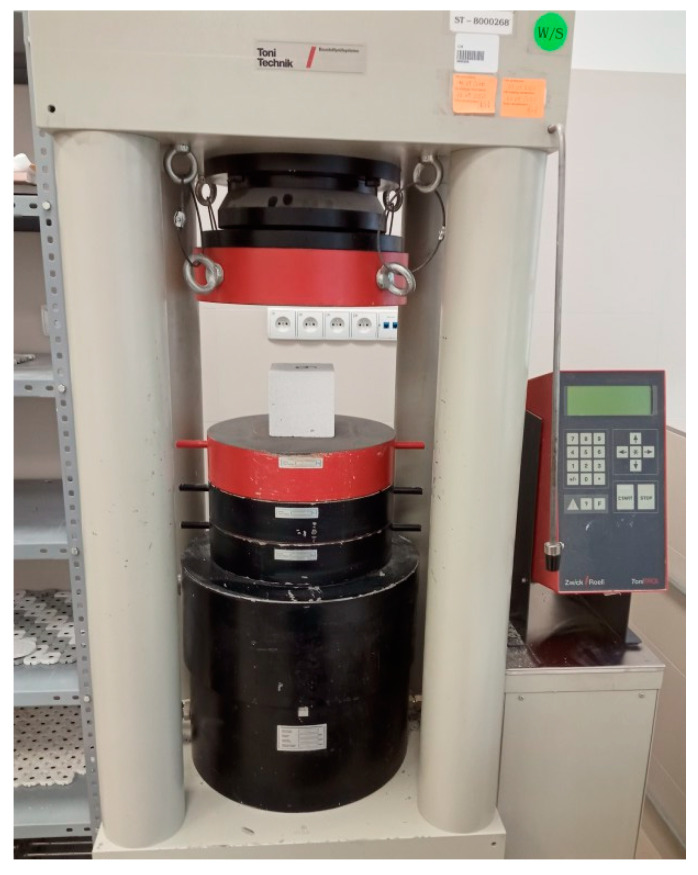
Toni Technik press machine.

Strength tests were performed using the destructive method by means of the Toni Technik strength press (Toni Technik, Berlin, Germany) (Figure 5), which involves crushing the sample until it ruptures (PN-EN 12390-3) [35]. The compression tests were performed using a 3000-kN capacity testing machine. The research was carried out at an air temperature of 21 ± 1 °C and humidity of 65 ± 5%, with the constant axial force rate of the samples in all of the experiments being approximately 5 kN/s. The result of the test is the highest value in MPa registered by the computer. Performing a concrete strength test allows the compressive strength of the concrete to be determined. The results give the opportunity to clearly determine whether the concrete mix meets the requirements for the strength specified in the documentation of the construction, which clearly translates into the safety of the entire building. Cubic samples with a side dimension of 100 mm were used to test the compressive strength of the concrete (PN-EN 206-1) [36].

#### 3.3.3. Computer Thomography Imaging Tests

**Figure 6:**
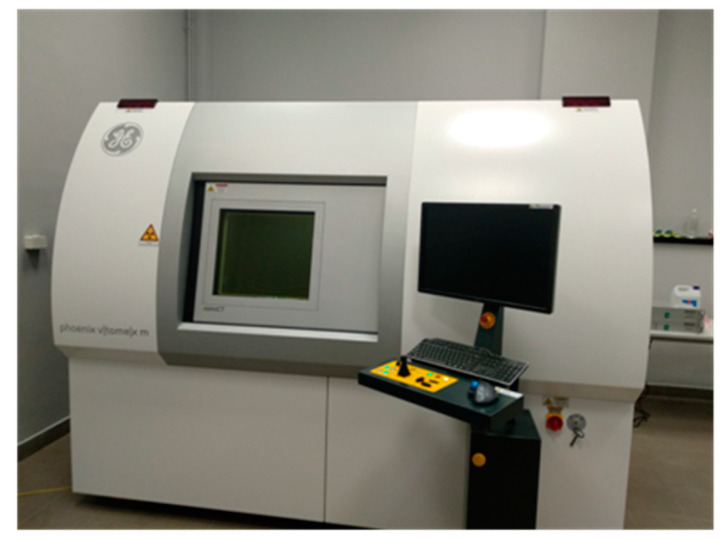
Tomograph from GE, model Vtomex m300.

Imaging tests were performed using a model Vtomex m300 General Electric (GE) tomograph (Waygate Technologies, Hürth, Germany) (Figure 6) with the detector GE DXR250 RT (X-ray tube) (Baker Hughes, Houston, TX, USA) with precision of less than 1 µm. The test consists of directing an X-ray beam onto a sample and registering its intensity on the detector. Creating an image involves measuring the absorption of radiation that passes through the sample. The concrete samples were placed on a rotating table and kept relatively stationary: the radiation cannon and detector, after the 360° full rotation of the sample, obtained a complete picture of the structure. The reconstruction was made using Volume Graphic’s VGStudio Max program.

The computed tomography method can be used to assess the microstructure of the binder and the number and shape of air pores in concrete. Plane images obtained on different depths of samples allow assessing the spatial distribution of the examined objects. The assessment of the distribution, size, and shape of pores has a decisive influence, among others to infer the durability of concrete. Pores formed accidentally as a result of the inappropriate selection of components or improper technology of mixing and laying concrete mix are harmful due to the strength and tightness of concrete and its frost resistance.

## 4. Results

### 4.1. Thermal Tests

**Figure 7:**
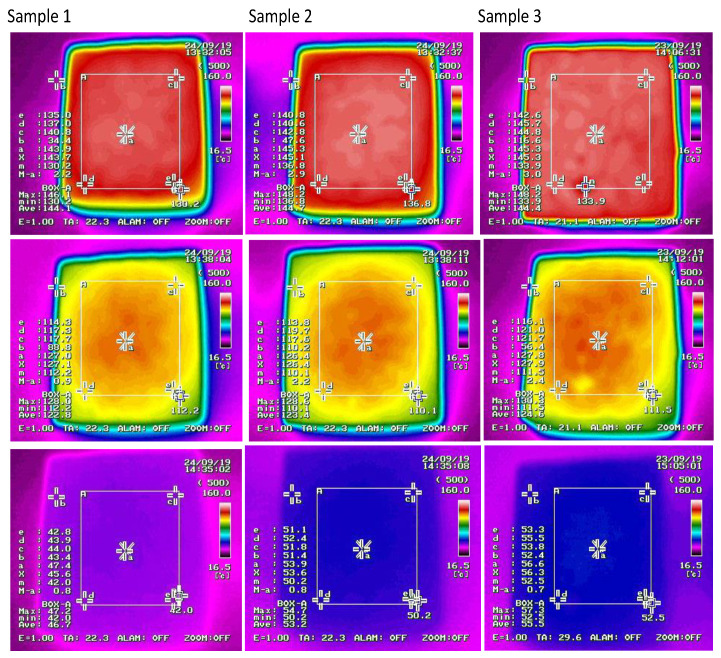
Sample photo from the thermal imaging camera of the tested concretes.

Table 3 summarizes the results of the temperatures obtained with regard to the cooling time. When analyzing the values of the temperature *ave* contained in Table 3, it was found that the fastest heat is given by concrete sample 1 (Figure 7) with 100% content of irregular aggregate—the direction of heat flow was parallel to the grain arrangement. On the other hand, concrete sample No. 3 cooled slowly, i.e., with a 100% regular aggregate content. The temperature difference in the final cooling phase was about 10 °C.

The temperature values read from the surface of the samples were used to calculate thermal parameters such as heat energy E, heat quantity ΔQ, heat power P, or volumetric heat capacity b. The energy consumed by the body during its heating, or given off during its cooling, is proportional to the product of the body mass m and the difference in body temperature before and after the heat transition ΔT. The measure of thermal energy emission is the thermal power P, which is determined by the amount of emitted energy E by the formula:

E = ΔQ = c m·ΔT, [J](1)

m—mass [kg]
ΔT—temperature difference before and after heat conversion [K]
c—specific heat of the tested concrete in a dry state [J/kg∙K] (calculated in Table 4).

Thermal power is determined by the formula:

P = E/t, [W](2)

t—emission time [s] = 3510 s.

Another parameter that characterizes materials in terms of their thermal properties is the volumetric heat capacity b. Its value is calculated as the product of specific heat c and density ρ of the material from which the solid is made:

b = c·ρ, [J/(m^3^K)]. (3)

An additional parameter that characterizes the efficiency of the material accumulation phenomenon is the maximum energy b_max_ that can be accumulated in a unit of volume of a given material, which can be described by the formula:

b_max_ = b·ΔT, [J/m^3^]. (4)

Table 5 shows additional parameters of the samples, necessary for thermal calculations. The results in Table 3 show that the sample of concrete with irregular aggregates cools faster than the sample of concrete with regular aggregate, with the final cooling temperature depending on the grain arrangement. Observations and measurements show that the special arrangement of irregular aggregate grains causes disturbed vertical movement of hot air and allows higher final temperatures of the material [38]. Irregular grains arranged laterally keep the temperature longer, creating a barrier to heat flow (final temperature 53.2 °C), while when arranged in parallel, they act as a kind of heat transfer (final temperature 46.7 °C) (Table 3). The maximum energy b_max_ that the material is able to accumulate and emit is 206.8 MJ/m^3^ for regular grains and 199.1 MJ/m^3^ and 191.8 MJ/m^3^ for irregular grains, respectively (Table 6). These values are characteristic for simple concretes [36,39].

### 4.2. Compressive Strength Tests

The w/c (water/cement) ratio and degree of hydration are among the most important factors of concrete strength. The w/c value is constant for all the samples and equal to 0.62. However, the shape and size properties of the aggregate particles that are used as fillers are also of major importance. The shape of aggregate particles mainly affects the demand for water and the amount of cement required for the workability of a given concrete mix [7].

**Figure 8:**
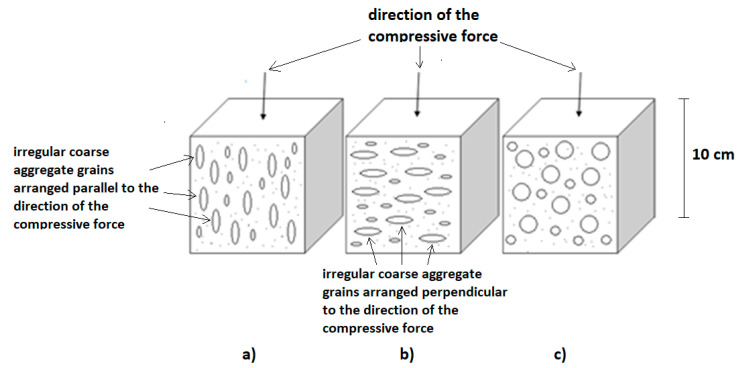
Type of specimens: containing 100% irregular grains (**a**) and (**b**), containing 100% regular grains (**c**). The research was conducted in accordance with the PN-EN 12390-3 standard [35].

Particles with an elongated shape require more water in the process of concrete production, and therefore the use of this type of aggregate decreases the workability of a concrete mix. On the other hand, smooth texture and rounded aggregate particles require less water to create concrete with an improved performance. The binding with cement can also be weakened due to water accumulation under relatively large surfaces of flat aggregate particles. For the same w/c ratio value, concrete with regular and broken aggregate particles has a higher compressive strength than concrete with irregular aggregates. The basic requirement for hardened concrete is its compressive strength. This property is closely related to the microstructure of the hardened cement, the strength of the aggregate, and the aggregate–cement contact area. The compressive strength of the concrete determines the grade and quality of the concrete. Concrete strength tests were carried out in accordance with the PN-EN 12390-3 [31] standard. Three types of concrete samples were tested, and six individual tests were performed for each type. The results are shown in Table 6. An average value of 49.2 MPa was obtained for the sample of concrete consisting of 100% regular particles, which was about 10% higher than for the other samples being tested (Table 7). It seems that an arrangement of irregular particles within the concrete is also important (Figure 8). 

A slightly higher average strength (44.6 MPa) was obtained when the direction of the force was parallel to the arrangement of particles (sample 1). For the perpendicular direction of breaking force (sample 2), the achieved value was 43.4 MPa. The results for samples with regular particles were also characterized with a lower standard deviation of strength (σ = 0.32) when compared to the results for samples with irregular particles—where the values for both types of samples were greater than 1. In addition, the perpendicular arrangement of irregular particles to the direction of the acting breaking force was also characterized by a higher standard deviation (σ = 1.12) than in the case of the sample with irregular particles arranged parallel to the direction of the force (σ = 1.03).

Based on these tests, it can be observed that the particle arrangement in the material, as well as their shape, has a significant impact on the mechanical strength of preplaced concrete. When knowing the direction of compression or the breaking force on the sample, the value of the concrete strength for a specific concrete element can be increased by using a suitable method of filling the mold. Based on the above test results, it can be concluded that elongated particles in the concrete mix should be avoided because they have a negative effect on concrete strength. In addition, flat and elongated particles of aggregate are difficult to compact, and thus, the workability of the concrete decreases and the water demand increases. Increasing the water content reduces the strength and durability of the concrete.

### 4.3. Investigations on the Internal Structure

**Figure 9:**
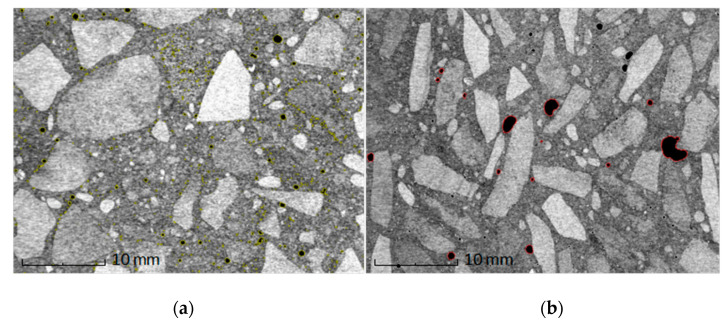
Cross-sections of samples of concrete with regular (**a**) and irregular (**b**) grains obtained by means of tomography. Pores that may affect the concrete quality are marked in black.

**Figure 10:**
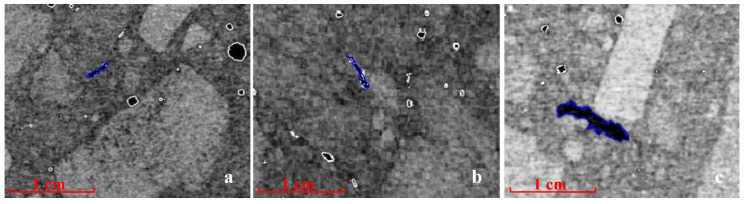
Tomography image of concrete samples with hydration cracks (scratches), which are market in blue: with regular grains (**a**,**b**) and with irregular ones (**c**).

**Figure 11:**
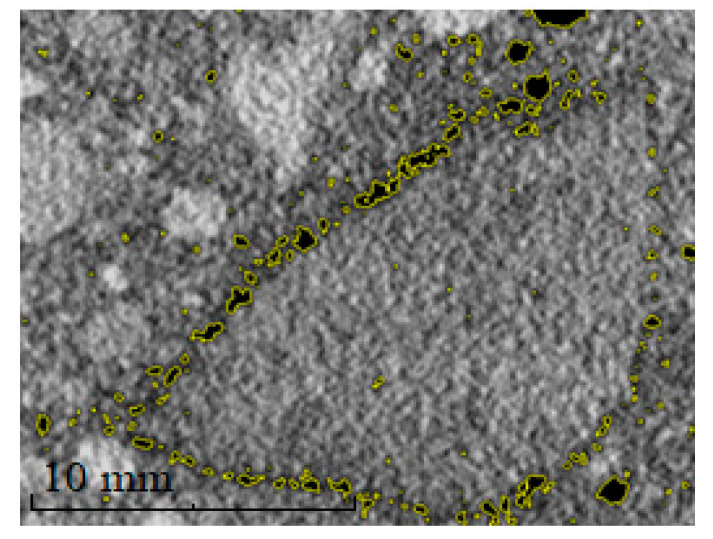
Image of aggregate grain with a visible debonding porosity—obtained using computer tomography.

Three factors influence concrete porosity: the porosity of the aggregate, the porosity of the cement mix, and the porosity of the contact area; the aggregate consists of approximately 70% of the concrete’s volume. The porosity of coarse aggregate is low in relation to the porosity of the cement mix and usually does not exceed a few percent. Moreover, the amount and structure of the cement mix’s pores directly translates into concrete strength and other useful properties such as frost resistance, water absorption, or corrosion resistance [40]. Thanks to the passing of radiation through the entire sample, X-ray tomography allows for measurements of even very complex structures with surfaces difficult to access, as well as invisible pores, voids, and gaps. Two types of air pores, round and elongated (Figure 9, Figure 10 and Figure 11), differing in the genesis of their origin, were observed on the CT images. The properties of concrete are influenced by the total pore volume and their size, as well as the uniformity of distribution and the shape of the pores.

It is assumed that small spherical pores not connected together of a size not larger than about 300 µm do not significantly affect the durability and properties of concrete. Continuous gap pores and large (over 300 µm) spherical pores cause the deterioration of strength parameters. An increase in the content of air voids by 1% causes a decrease in compressive strength by approximately 5% [41]. Figure 9 presents the structural image of the concrete samples with regular and irregular particles. The sample with irregular particles is characterized by a significantly higher content of spherical large pores, which are located at the boundary of the aggregate–binder phase. Such porosity arises due to the accidental introduction of air into the mix. The overall porosity, measured by the hydrostatic method, was on average 6.82% for the samples of concrete with irregular grains and 3.78% for the samples of concrete with regular grains. Irregular particles, due to their elongated shape, are more difficult in operations of concrete mixing and compaction, where the turning of the particles causes a turbulent movement in the cement mix and blocks air between the aggregate and cement. This fact has a direct impact on the strength of the samples (Table 7).

Slit-like pores are formed as a result of the evaporation of demand water in the concrete mix in processes of clinker phase hydration. During the formation of concrete, significant amounts of heat are emitted, which results in thermal stresses and an increase in the vapor pressure of excess demand water. The risk of thermal gaps forming is highest in the initial hydration stage, when a fresh concrete has a lower strength. In Figure 10, hydration cracks are visible, with the largest of them being observed in the concrete sample with irregular aggregate particles. This is due to the increased demand for water if this type of aggregate is used. Coarse and regular aggregate makes it possible to reduce the amount of cement mix, and thus in turn reduce the risk of hydration scratches [1].

Figure 11 shows an aggregate particle with visible debonding porosity. In other words, these are the pores formed on the border between the aggregate and cement. Such pores with a diameter below 1 mm are formed due to dust and the impurities of the aggregate surface. Crystals of clay minerals are particularly unfavorable in this respect. These minerals are characterized by a large specific surface and layered structure, which promotes swelling and the adsorption of various chemical compounds, including the carboxylates that are part of the cement modifiers [42,43,44]. These properties cause the failure of the cement matrix to bind with an aggregate, which is why it is extremely important to use washed aggregate when producing concrete mixes.

## 5. Conclusions

Based on the results of this study, the following conclusions can be drawn:

1. Regular aggregates have a positive effect on concrete quality in terms of its durability, as well as in terms of its improved thermal properties and lower number of large pores. The utilization of regular aggregates improves the quality of construction materials.
2. During the preparation of the concrete mixes for the tests, variations in flow, and thus in concrete consistency, were observed. The flow was higher (175 mm) for the sample with regular particles when compared to the sample with irregular ones (150 mm). The consistency of the mix was thinner, which confirms the lower water and cement demands of the mix.
3. Thermic tests carried out with the use of a thermal camera showed that the concrete sample with irregular particles of aggregate was cooling faster than the sample with regular particles. Moreover, the final temperature of cooling depends on the particle arrangement in the sample. Irregular particles arranged transversely keep the temperature longer, creating a barrier for the heat flow (final temperature 53.2 °C), while the parallel arrangement of aggregate particles in a sample act as a heat transporter (the final temperature was 46.7 °C). Maximum energy b_max_, which could be cumulated and emitted by the material, was 206.8 MJ/m^3^ for regular particles and 199.1 MJ/m^3^ and 191.8 MJ/m^3^ respectively for irregular particles. These values are typical characteristics for ordinary concretes.
4. On the basis of the strength test results, it could be observed that the manner of aggregate particle arrangement in the material, as well as their shape, significantly influences the mechanical strength of concrete. By knowing the direction of the compressive force acting on a sample, it is possible to increase the value of concrete strength with an appropriate method of laying the concrete mix, which depends on its intended use.
5. Concretes with 100% regular particles (six samples with 100% regular grains) obtained about a 10% higher strength, equal to 49.2 MPa, when compared to the remaining samples with irregular particles. The standard deviation results were lower for the concretes with regular grains when compared to the samples with irregular particles.
6. Tests of the internal structure of the concretes using a tomograph showed that the concrete with irregular particles is characterized by a significant detachment porosity and size of hydration gaps, as well as a much higher content of large spherical pores (with a size of over a few millimeters), which are located at the aggregate–cement matrix phase boundaries. This fact has a direct impact on the strength of the samples and the increased demand for mixing water when using this type of aggregate.
7. The aim of this investigation was to show the impact of the coarse particle size fraction aggregate (12–14 mm) on the selected parameters of the concrete mix and hardened concrete. Subsequent research will focus on testing regular and irregular aggregates in concretes with a constant and optimal particle size composition. The cost of producing regular grains and irregular aggregates, regardless of the regularity of the grains, is the same. While maintaining the same parameters of concrete, it is possible to reduce the amount of cement in the concrete mix by using aggregate of an appropriate shape. Detailed cost analyses will be presented in a separate article.

## Figures and Tables

**Table 1:** Average values and the standard deviation of density and water absorbability obtained from the measurements.

Product	Density [Mg/m^3^]	Standard Deviation [Mg/m^3^]	Water Absorbability [%]	Standard Deviation [%]
**Regular grain aggregate**	2.65	0.05	3.24	0.24
**Irregular grain aggregate**	2.64	0.02	3.43	0.99

**Table 2:** Concrete mix composition.

Ingredients for 1 m^3^ of Mix	Weight [kg]
CEM I 42.5 R	380
Sand	980
Dolomite	980
Water	240

**Table 3:** Temperature of the tested samples with regard to the cooling time.

Time [s]	Concrete Sample Type		
	1 Irregular Grains 100%, Direction of Heat Flow Parallel to the Grain Arrangement	2 Irregular Grains 100%, Direction of Heat Flow Perpendicular to the Grain Arrangement	3 Regular Grains 100%
Temperature °C			
0	144.1	144.7	144.4
30	141.5	141.5	141.1
60	138.9	137.7	139.4
90	135.2	135.6	137.0
150	133.6	132.5	134.2
210	129.6	129.5	131.2
330	122.8	123.4	124.6
510	110.5	113.4	114.6
1110	80.4	82.2	89.9
1710	66.9	68.8	73.7
2610	57.6	54.4	58.6
3510	46.7	53.2	55.5

**Table 4:** Calculated specific heat of the tested concrete mixes.

Specific Heat	[J/kg∙K]
Concrete binder according to PN-EN ISO 12524 [37]	1000
Dolomite:	
CaMg(CO_3_)_2_	943
CaO	750
MgO	924
CO_2_	1073
Sand: SiO_2_	742
Specific heat of tested sample	881

**Table 5:** Additional parameters of the tested concretes.

Sample	m [kg]	ρ [kg/m^3^]	ΔT [K]
1 Irregular grains 100% a	0.980	2410	97.4
2 Irregular grains 100% b	1.030	2470	91.5
3 Regular grains 100%	1.045	2450	88.9

**Table 6:** Selected thermal parameters of the concrete.

Sample	ΔQ [J]	P [W]	b [MJ/(m^3^K)]	b_max_ [MJ/m^3^]
1 Irregular grains 100% a	84 093	23.96	2.123	206.8
2 Irregular grains 100% b	83 030	23.66	2.176	199.1
3 Regular grains 100%	81 845	23.32	2.158	191.8

**Table 7:** Compressive strength of the concrete samples according to the PN-EN 12390-3 standard.

Type of Sample	Average Compressive Strength of Concrete [MPa]	Standard Deviation [MPa]	Standard Deviation [%]
1 Irregular grains 100%, a	44.6	1.03	2.31
2 Irregular grains 100%, b	43.4	1.12	2.58
3 Regular grains 100%	49.2	0.32	0.65
